# Outcome and death risk of diabetes patients with Covid-19 receiving pre-hospital and in-hospital metformin therapies

**DOI:** 10.1186/s13098-021-00695-8

**Published:** 2021-07-13

**Authors:** Rodrigo Esaki Tamura, Said Muhammad Said, Leticia Mussin de Freitas, Ileana Gabriela Sanchez Rubio

**Affiliations:** 1 Department of Biological Sciences, Federal University of São Paulo, Rua Pedro de Toledo 669, 11º Andar, Diadema, SP Brazil; 2 Thyroid Molecular Sciences Laboratory, Federal University of Sao Paulo, São Paulo, SP Brazil; 3 Laboratory of Cancer Molecular Biology, Federal University of Sao Paulo, São Paulo, SP Brazil; 4 Santa Catarina Hospital, São Paulo, Brazil

**Keywords:** COVID-19, Diabetes, Metformin, Brazil, Survival

## Abstract

**Background:**

COVID-19 has stroke Brazil harshly, deaths by COVID-19 in Brazil represent almost 13% of the total deaths by COVID-19 in the world, even though Brazilian population represents only 2.6% of the world population. Our aim in this study was to evaluate death and intubation outcomes and risk factors associated with COVID-19, and treatment options focusing on diabetes patients and the use of metformin pre-admission and during hospitalization.

**Methods:**

In this Brazilian single-center study we evaluated 1170 patients hospitalized due to COVID-19. Diabetes patients (n = 188) were divided based on their use of pre-hospital and in-hospital metformin (non-met-group and met-group).

**Results:**

In the total cohort most comorbidities were risk factors for orotracheal intubation and death. The use of chloroquine/hydroxychloroquine was significantly associated with increased death and intubation risk in uni- and multivariate analysis. Diabetes patients showed worst clinical feature compared with non-diabetes patients. In-hospital non-met-group had increased mortality (20.5%) compared to met-group (3.5%) (p = 0.0002) and univariable cox proportion hazard regression indicated in-hospital metformin reduced mortality (HR = 0.325, p = 0.035). Patients that used pre-hospital metformin showed lower severity parameters at hospital admission. (met-group: 2.45 ± 2.5; non-met-group: 4.25 ± 3.4). In all the groups older patients showed more severe clinical conditions and high risk of death and intubation.

**Conclusion:**

Even though this is a single-center study, results from other reports have shown a similar trend, indicating that patients that used metformin during hospitalization have a better prognosis and reduced risk of death.

**Supplementary Information:**

The online version contains supplementary material available at 10.1186/s13098-021-00695-8.

## Background

The COVID-19 pandemic is caused by SARS-CoV-2, a member of the Betacoronavirus genus of the Nidovirales order, its single stranded positive polarity RNA encodes non-structural and structural proteins, one important structural protein is the Spike, which mediates recognition of the ACE2 cell receptor. The non-structural proteins play pivotal role in cell regulation, that impacts host response to viral infection [1]. Data from the World Health Organization (WHO) points to over 178 million confirmed cases and 3,864,180 deaths until June, 21st 2021 (https://covid19.who.int/). Deaths by COVID-19 in Brazil has reached the number of 501,825 on June, 21st 2021 (https://covid.saude.gov.br/).

Diabetes is one of the main risk factors for COVID-19, promoting a harmful pro-inflammatory state [2]. Hyperglycemia has been reported in 51% of the COVID-19 patients [3] and is concurrent with increase in inflammatory mediators and aberrant glycosylation of ACE2 receptor, which may favor SARS-Cov-2 infection [4]. The Chinese Center for Disease Control and Prevention indicated an overall case fatality rate (CFR) of 2.3%, while CFR was elevated to 7.3% for diabetes patients [5]. Prevalence of diabetes in COVID-19 patients ranges from 5 to 20% in different studies. Diabetes is also associated with high risk of severe to critical illness of 14 to 32% of the COVID-19 patients [6].

Several drugs have been analyzed for drug repurposing for COVID-19 treatment. Metformin is commonly used to treat diabetes patients. In the beginning of the COVID-19 pandemic some hospital protocols recommended discontinuation of metformin for COVID-19 patients [7]. It was suggested that metformin could induce lactic acidosis and therefore should be discontinued [8]. Even tough metformin had a positive correlation with acidosis and lactic acidosis, it showed no significant difference in mortality [9]. Only one preliminary report indicated an increased risk for life-threatening complications due to metformin use [10]. Afterwards it had been suggested for the FDA to change metformin on-label use as adjuvant therapy against COVID-19 in obese, elderly and diabetes patients [11]. Different reports indicated a positive correlation of survival with use of metformin prior to admission [12–17], or during hospitalization [18–22], while others have not observed any correlation with time of hospitalization, severity or death by COVID-19 patients that used metformin in admission [23, 24].

In this report we investigated 1,170 COVID-19 positive patients that were admitted for hospitalization in a Brazilian single-center, the Santa Catarina Hospital—São Paulo. We aimed to characterize the clinical features of these patients, to evaluate risk factors and to compare the outcome of diabetes and non-diabetes patients and of metformin users and non-users.

Our results indicate that older patients have worse clinical features, patients under metformin therapy had reduced COVID-19 severity at admission and the use of metformin during hospitalization by diabetes patients reduce the risk of death, while chloroquine/hydroxychloroquine (CHLO/HCQ) were associated with increased risk of death and intubation.

### Design

This is a monocentric retrospective study of the COVID-19 patients hospitalized at the Santa Catarina Hospital- São Paulo- Brazil. Demographic and clinical variables were collected from hospital administrative records from 1244 patients that were hospitalized from March 10 to November 13, 2020 with COVID diagnosis confirmed on the basis of a positive nasopharyngeal SARS-CoV-2 RT-PCR test. Pregnant women and patients without outcome (ex. transferred to other hospitals) were excluded from the study. From the remaining 1170, we selected 1083 patients aged 29 years old or higher and grouped in diabetic (n = 188) and non-diabetic (n = 895). The cut off of 29 was chosen because no diabetes patient was below this age. Diabetes was established through the diagnosis in medical records or self-reported, confirmed during hospitalization. The diabetic group was also divided into metformin users (met-users) and non-metformin users (non-met-users), considering those that received metformin therapy pre-hospitalization (met-users n = 116; non-met-users n = 72) or during hospitalization at least one day (met-users n = 115; non-met-users n = 73) or not. Patients were also grouped by age (0–28; 29–59; ≥ 60) for some analysis.

Demographic and clinical data included age, sex, total time of hospitalization, time of stay in intensive care unit (ICU), time of stay in nursery, time of mechanical ventilation, orotracheal intubation, use of prisma hemodialysis machine, prone position, and the hospital medications: vasoactive drug, and chloroquine/hydroxychloroquine (CHLO/HCQ) that comprise the same variable. We also included the comorbidities: smoking and former smoking, diabetes, hypertension, cardiovascular disease, chronic obstructive pulmonary disease (COPD) and pneumonia, neurological disease, neoplasia and immunosuppression, thyroid disease, chronic kidney disease (CKD), dyslipidemia and asthma. For the diabetic group it was also included: type of diabetes, body mass index and use of metformin therapy prior to hospitalization and the in-hospital medications metformin, insulin and dexamethasone/prednisolone (both in the same variable).

At hospital admission the clinical severity of patients was graded based on the National Early Warning Score (NEWS) that considered respiratory rate, oxygen saturation, oxygen supplementation, systolic blood pressure, pulse rate, level of consciousness and body temperature of adult patients, or on the Pediatric Early Warning Score (PEWS) that consider behavior, cardiovascular status, respiratory status of children. This study was approved by the local Ethical Committee (CAAE 43014721.7.0000.5505).

### Statistics analysis

Continuous variables were expressed as mean ± SD or median [max–min] and categorical variables as number and percentage (%). Differences between groups were analyzed using Student’s T-test, Mann–Whitney test, Kruskal Wallis Tests or ANOVA for continuous variables, and by Chi^2^ or Fisher’s exact test for categorical variables. Logistic regression analysis was used in the univariate and multivariate analysis to determine odds ratio and 95% confidence interval [OR (CI 95%)] for factors associated with the primary outcomes in-hospital mortality and orotracheal intubation, the results were not adjusted for multiple tests. Risk factors associated with in-hospital deaths were also assessed using a multivariable Cox proportional hazards regression model. Kaplan Meier method was used to compare the cumulative probability of in hospital death. The analyses were based on non-missing data. Statistical significance was defined as p < 0.05 and a two-side α was considered in the analysis. All statistical analyses were performed using IBM SPSS Statistics software version 25.0.

## Results

### Demographic and clinical characteristics of the cohort

A total of 1,170 patients with COVID-19 were included in this study, 47.2% were women and 52.8% were males with mean age 51.8 ± SD 18.4. The most common comorbidities were hypertension (31.5%), diabetes (16.19%), dyslipidemia (14.6%) and obesity (10.4%). Among those patients 4.2% (n = 49) died during hospitalization. Univariable logistic regression showed that age and mainly, elderly of 60 years old or higher (≥ 60 group) was associated with high risk of in-hospital death [OR 119,11 (16.37—866,54.3)] and with orotracheal intubation [OR 9.24 (2.22–38.51)] when compared with the 29–59 group. Considering the comorbidities, diabetes, cardiopathy hypertension, COPD/pneumonia, neurological disease, CKD and dyslipidemia were also death risk factors. All of the in-hospital clinical characteristics except the time of stay in nursery were associated with increased risk of death. Furthermore, all comorbidities, except asthma, and all clinical features, except time of mechanical ventilation and the use of prisma were risk factors associated with orotracheal intubation (Table 1).

**Table 1:** Demographic and clinical characteristics of all patients hospitalized with Covid-19 and univariate in-hospital risk factors associated with in-hospital mortality and orotracheal intubation

Clinical features	All patients (n = 1170) number (%) median (max–min)	Mortality risk factors OR (CI 95%)	Orotracheal intubation risk factor OR (CI 95%)
Gender			
Man	618 (52.8%)	1.078 (0.608–1.91)	0.669 (0.43–1.03)
Woman	552 (47.2%)		
**Age, years**	51.8 ± 18.4	**1.14 (1.11–1.17)**	**1.05 (1.04–1.07)**
Age class			
< 29	87 (7.4%)		
29–59	713 (60.9%)		
≥ 60	370 (31.6%)	119.11 (16.37–54.3)*	9.24 (2.22–38.51)*
Clinical outcome			
Mortality	49 (4.2%)	–	–
Mortality by age class			
< 29	0 (0%)	–	–
29–59	1 (0.1%)	–	–
≥ 60	48 (13%)	–	–
Comorbidities			
Diabetes (type I or II)	188 (16.1%)	**3.57 (1.96–6.48)**	**6.08 (3.9–9.47)**
Obesity	122 (10.4%)	0.97 (0.38–2.51)	**2.03 (1.15–3.56)**
Cardiopathy	112 (9.66%)	**6.34 (3.42–11.77)**	**3.14 (1.84–5.34)**
Hypertension	368 (31.5%)	**5.92 (3.14–11.14)**	**4.00 (2.59–6.2)**
COPD/pneumonia	62 (5.3%)	**6.95 (3.41–14.33)**	**2.68 (1.34–5.34)**
Smoking/former smoking	43 (3.6%)	2.46 (0.85–7.2)	**1.16 (0.41–3.33)**
Neurological disease	70 (6.05%)	**7.6 (2.072–24.93)**	**3.14 (1.67–5.88)**
CKD	28 (2.4%)	**8.73 (3.52–21.67)**	**8.13 (3.69–17.93)**
Thyroid disease	111 (9.5%)	1,63 (0.71–3.72)	**2.23 (1.06–4.71)**
Dyslipidemia	171 (14.6%)	**3.02 (1.64–5.62)**	**1.59 (0.85–2.97)**
Neoplasia/immuno	57 (4.9%)	2.34 (0.89–6.14)	**2.83 (1.76–4.57)**
Asthma	51 (4.4%)	0.45 (0.06–3.3)	3.18 (0.43–23.3)
In-hospital clinical characteristics			
NEWS/PEWS	1 (0–13.2)	**1.5 (1.36–1.63)**	**1.38 (1.28–1.48)**
Time of stay in ICU	0 (0–107)	**1.082 (1.058–1.11)**	**1.42 (1.34–1.5)**
Time of stay in nursery	5.0 (0–61)	0.95 (0.88–1.02)	**1.09 (1.06–1.12)**
Time of hospitalization	6.0 (1–112)	**1.047 (1.03–1.06)**	**1.18 (1.15–1.21)**
Time of MV	0 (0–66)	**1.16 (1.12–1.21)**	NS
Orotracheal intubation	94 (8%)	**35.2 (18.42–67.65)**	–
Prisma	24 (2.4%)	**44.162 (19.36–100.74)**	NS
Prona	18 (1.5%)	**6.93 (2.19–21.88)**	**233.8 (30.7–1280.23)**
Vasoactive drugs	72 (6.2%)	**32.15 (16.93–61.044)**	**690.46 (234.27–2034.92)**
CLHO/HCQ	76 (6.5%)	**1.11 (1.03–1.21)**	**22.83 (5.2–100.17)**

NS, not significant, Neoplasia/immune, neoplasia/immunosuppression; Time of MV, Time of mechanical ventilation; COPD, chronic obstructive pulmonary disease; CKD, chronic kidney disease; NEWS/PEWS, National Early Warning Score/Pediatric Early Warning Score; Prisma, prisma hemodialysis machine; Prona, prone position; CLHO/HCQ, Chloroquine/hydroxychloroquine; *compared with 29–59 group. Bold: p < 0.05

In the multivariate analysis when age, gender and all comorbidities were included in the analysis, age was associated with the risk of death and orotracheal intubation, and Diabetes Mellitus, obesity and CKD remained independent risk factors for orotracheal intubation (Table 2). Considering all the in-hospital clinical variables, the use of prisma, NEWS/PEWS and the use of CHLO/HCQ were in-hospital death risk factors and NEWS/PEWS and the use of CHLO/HCQ were orotracheal intubation risk factors.

**Table 2:** Multivariable analysis of in hospital mortality and orotracheal intubation in the complete cohort (n = 1170)

Characteristic	Mortality risk factors OR (CI 95%)	Orotracheal intubation risk factors OR (CI 95%)	Covariables included in the logistic analysis
Age Diabetes Mellitus Obesity CKD	1.142 (1.03–1.18) NS NS	1.042 (1.02–1.06) 3.29 (1.99–5.47) 2.158 (1.13–4.11) 3.1 (1.22–7.82)	Age and gender and all comorbidities
Prisma NEWS/PEWS CLHO/HCQ	12.06 (1.31–110.81) 1.34 (1.06–1.7) 10.46 91.23–88.52)	NS 1.34 (1.06–1.69) 10.16 (1.2–86.02)	Orotracheal intubation, prona, prisma, NEWS/PEWS Vasoactive drug and CLHO/HCQ

NS, not significant; COPD, chronic obstructive pulmonary disease; CKD, chronic kidney disease; NEWS/PEWS, National Early Warning Score/Pediatric Early Warning Score; Prisma, prisma hemodialysis machine; Prona, prone position; CLHO/HCQ, Chloroquine /hydroxychloroquine

These results clearly indicate that patients with worse clinical features at admission or that needed more invasive therapies, had also increased chance for intubation and death risk. Also, the use of CHLO/HCQ instead of having a protective potential, was associated with a significant increase of both the need for mechanical ventilation and death risk (Table 2). However, there was no direct correlation between cardiopathy and use of CHLO/HCQ with death risk (p = 1.151), despite a death rate of 50% of cardiac patients that received CHLO/HCQ and only 24.2% for the patients that didn’t receive these drugs.

### Demographic and clinical characteristics of the diabetes patients of the cohort

At all 188 patients, 16% of the studied cohort, had the diagnosis of diabetes at hospital admission. In this study type I and II diabetes patients were included (type I n = 5; type II n = 183), as use of metformin was the focus and patients from both types have used metformin. The youngest patient of this group was 29 years old, thus for comparison, only patients over 29 years old (n = 895) were included in the non-diabetic group. The diabetic group showed higher frequency of men compared with non-diabetic (62.8% vs 51.3% p = 0.005), higher median age (64.59 + 14.9 vs 52.44 + 15.6, p = 0.005) and higher frequency of patients over 60 years old (61.7% vs 28.4%, p = 0.000). All the comorbidities, except neurological disease, thyroid disease, neoplasia/immunosuppression, and all the in-hospital clinical characteristics, except the use of CHLO/HCQ, were significantly more frequent in diabetes patients when compared with non-diabetes. In agreement with these worse clinical features, the diabetes patients also had an increased mortality compared with non-diabetes (10.1% vs 3.4% p = 0.000) and almost all the dead patients of diabetic and non-diabetic groups were ≥ 60 years old (Additional file 1: Table S1).

We also compared the diabetes patients by age class (≥ 60 group vs 29–59 group). The ≥ 60 group showed high mortality (15.5% vs 1.4%, p = 0.002), high rate of neurological disease; hypertension; cardiopathy, COPD/pneumonia), use of vasoactive drugs; orotracheal intubation and use of prisma. All smokers or former smokers were in the ≥ 60-group. This group showed higher scores of clinical severity at admission (NEWS), longer hospital stay and longer stay in ICU, longer time of in-hospital metformin and increased time of mechanical ventilation compared with 29–59 group (Additional file 1: Table S2). Between patient of the ≥ 60 group, high mortality was observed in patients with orotracheal intubation (88.9% vs 19.8%, p = 0.000); that used prisma (50% vs 4.2%, p = 0.000) or vasoactive drug (77.7% vs 18.8%, p = 0.000); cardiopathy associated (66,7% 22.4%, p = 0000); suffering from COPD/pneumonia (33.3% vs 2.8%, p = 0.013), neurological disease (27.8% 7.1%, p = 0.02) or CKD (27.8% vs 4.1%, p = 0.004), and who received CHLO/HCQ (47.1% vs 10.3%, p = 0.001).

### Metformin therapy in diabetes patients

Next, we investigated the outcome of metformin therapy during hospitalization in the diabetes patients. Overall, we observed a significative reduction of mortality in the met-group when compared with non-met-group (3.5% vs 20.5%, p = 0.0002). As metformin is an oral drug, orotracheal intubation interrupts the use of metformin. Therefore, considering only patients that did not needed orotracheal intubation during hospitalization, it was observed a tendency of increased number of deaths in the non-met patients compared with met-patients (5.6% vs 0%, p: 0.053). Considering the comorbidities, the in-hospital metformin therapy was associated only with reduced hypertension and CKD. When the clinical characteristics were investigated, the metformin users only showed reduction in the in-hospital time of insulin therapy and frequency of patients that used prisma [3 days (1–42) vs 15.5 days (1–98), p = 0.019]; (3.6% vs 15.1%, p = 0.011), respectively (Table 3). Which indicate that metformin therapy could improve renal condition of the diabetes patients. But, the benefit of metformin therapy was not confined by improvement of clinical features and was also not influenced by age, comparing the met and non-met groups there is no difference in NEWS or age (Table 3).

**Table 3:** Clinical characteristics of diabetes patients, comparison between in-hospital metformin users and non-users

	Non-met-group (n = 73) Number (%)	Met-group (n = 115) Number (%)	*P*^a^
Gender			
Female	28 (38.4%)	42 (36.5)	0.80
Male	45 (61.6%)	73 (63.5%)	
Age	67 (29–95)	63 (37–92)	0.26
Age class (years)			
29–59	25 (34.2%)	47 (40.9%)	0.44
≥ 60	48 (65.8%)	68 (59.1%)	
Clinical outcome			
Mortality	15 (20.5%)	4 (3.5%)	**0.0002**
Mortality by gender			
Female	9 (81.8%)	2 (50%)	1
Male	6 (75.0%)	2 (50%)	
Mortality by age class			
29–59	1 (6.7%)	0 (0%)	
≥ 60	14 (19.18%)	4 (3.48%)	**0.0006**
Comorbidities			
Obesity	11 (15.1%)	20 (17.4%)	0.26
Cardiopathy	19 (26.0%)	22 (19.1%)	0.28
Hypertension	57 (78.1%)	68 (59.1%)	**0.007**
COPD/pneumonia	8 (11.0%)	9 (7.8%)	0.46
Smoking/former smoking	4 (5.5%)	11(9.6%)	0.41
Neurological disease	7 (9.6%)	6 (5.2%)	0.25
CKD	9 (12.3%)	2 (1.7%)	**0.004**
Thyroid	9 (12.3%)	15 (13%)	0.89
Dyslipidemia	33 (45.2%)	37 (32.2%)	0.07
Neoplasia/immuno	5 (6.8%)	7 (6.1%)	0.83
Asthma	1 (1.4%)	3 (2.6%)	0.56
BMI	29.42 ± 5.14	29.24 ± 5.28	0.16
In-hospital clinical characteristics			
NEWS	2 (0–11)	2 (0–9.3)	0.63
Time of stay in ICU	3 (0–94)	2 (0–41)	0.27
Time of stay in nursery	6 (0–39)	7 (0–58)	0.54
Total hospitalization time	10 (3–100)	11 (2–65)	0.57
Time of MV	0.0 (0–63);	0.0 (0–26)	0.09
In-hospital metformin therapy	–	115 (100%)	–
In-hospital max dose of metformin	–	1249.13 ± 578.7	–
In-hospital time of metformin therapy	–	8.87 ± 9.24;	–
Pre-hospital metformin therapy	19 (26%)	97 (84.3%)	**0.000**
Pre-hospital metformin daily dose	1235.3 ± 597.22	1127.01 ± 629.21	0.51
In-hospital insulin therapy	18 (24.7%)	43 (37.4%)	0.08
In-hospital time of insulin therapy^a^	15.5 (1–98)	3 (1–42)	**0.019**
Orotracheal intubation	20 (27.4%)	25 (22.5%)	0.45
Prisma	11 (15.1%)	4 (3.6%)	**0.011**
Prone	4 (5.5%)	3 (2.7%)	0.4
Vasoactive drugs	19 (26%)	18 16.2%	0.1
CLHO/HCQ	12 (16.9%)	13 (11.3%)	0.27
Dexamethasone/prednisolone	65 (89.0%)	104 (90.4%)	0.75

COPD, chronic obstructive pulmonary disease; CKD, chronic kidney disease; NEWS/PEWS, National Early Warning Score/Pediatric Early Warning Score; Prisma, prisma hemodialysis machine; Prona, prone position; Neoplasia/immune, Neoplasia/immunosuppression; BMI, body mass index; Time of MV, Time of mechanical ventilation; In-hospital max dose of metformin: In-hospital maximum dose of metformin; CLHO/HCQ, Chloroquine/hydroxychloroquine. ^a^time in days. Bold: p < 0.05

Another important aspect to be considered is that patients who used metformin before hospitalization were checked-in with reduced clinical severity (NEWS) (2.45 + 2.5% vs 4.25 ± 3.43%, p = 0.001). These patients also showed reduced cardiopathy, hypertension, COPD/pneumonia and neurological disease, and as expected most patients that received pre-hospital metformin also received in-hospital metformin 83.6% compared with 25% that did not receive metformin pre-hospitalization (p = 0.000) (Table 4). Reduced mortality was observed between pre-hospital met-users and not-met-users (5.2% vs 18.1%, p = 0.004), however, these values were influenced by in-hospital metformin therapy. Comparing only the patients that did not receive in-hospital metformin, there was no differences in mortality rates between these groups (21.1% vs 20.4%, p = 0.9). Indicating that even though pre-hospital metformin use may improve clinical parameters at admission, its continuous use during hospitalization is essential. Indeed, patients that used pre-hospital metformin therapy but interrupted the treatment during hospitalization showed higher mortality than those that continued metformin therapy (21.1% vs 2.1%, p = 0.001).

**Table 4:** Clinical characteristics of patients that received pre-hospital metformin therapy and risk of death and orotracheal Intubation

	Non-met-group (n = 72) Number (%);	Met-group (n = 116) Number (%);	P*
Gender			
Female	26 (36.1%)	44 (62.9%)	0.8
Male	46 (39.9%)	72 (61.0%)	
Age	68.62 ± 17.27	62.09 ± 15.13	**0.006**
Age class (years)			
29–59	21 (29.2%)	51 (44%)	
≥ 60	51 (70.8%)	65 (56%)	**0.042**
Clinical outcome			
Mortality	13 (18.1%)	6 (5.2%)	**0.004**
Mortality by gender			
Female	6 (23.1%)	2 (4.5%)	
Male	7 (15.2%)	4 (5.6%)	1
Comorbidities			
Obesity	15 (20.8%)	16 (13.8%)	0.206
Cardiopathy	24 (33.3%)	17 (25.3%)	**0.003**
Hypertension	55 (76.4%)	70 (60.3%)	**0.023**
COPD/pneumonia	11 (15.3%)	6 (5.17%)	**0.019**
Smoking/former smoking	6 (8.3%)	9 (7.8%)	0.888
Neurological disease	9 (12.5%)	4 (3.4%)	**0.017**
CKD	8 (11.1%)	3 (2.6%)	**0.015**
Thyroid	10 (13.9%)	14 (12.1%)	0.716
Dyslipidemia	31 (43.1%)	39 (33.6%)	0.193
Neoplasia/immuno	3 (4.2%)	9 (7.8%)	0.327
Asthma	2 (2.8%)	2 (1.7%)	0.638
BMI	29.49 ± 4.96	29.203 ± 5.38	0.714
In-hospital Clinical characteristics			
NEWS	4.25 ± 3.43	2.45 ± 2.51	**0.001**
Time of stay in ICU	4 (0–58)	1 (0–94)	0.157
Time of stay in nursery	6 (0–58)	7 (9–28)	0.285
Total hospitalization time	11 (2–72)	10 (2–100)	0.119
Time of MV	0 (0–50)	0 (0–63)	0.168
In-hospital metformin therapy	18 (25%)	97 (83.6%)	**0.000**
In-hospital max dose of metformin	1158.33 ± 403.75	1265.98 ± 605.77	0.349
In-hospital time of metformin therapy	6 (1–22)	6 (1–56)	0.554
Pre-hospital metformin therapy	0	116	–
Pre-hospital metformin daily dose	–	1144.71 ± 622.55	–
In-hospital insulin therapy	25 (34.7%)	36 (31.0%)	0.600
In-hospital time of insulin therapy	7 (1–55)	5 (1–98)	0.844
Orotracheal intubation	22 (30.6%)	23 (20.5%)	0.123
Prisma	10 (13.9%)	5 (4.5%)	**0.023**
Prone	4 (5.6%)	3 (2.7%)	0.312
Vasoactive drugs	21 (29.2%)	16 (14.3%)	**0.014**
CLHO/HCQ	10 (13.9%)	5 (4.5%)	**0.023**
Dexamethasone/prednisolone	67 (93.1%)	102 (87.9%)	0.257

NS, not significant, Neoplasia/immune, Neoplasia/immunosuppression; BMI, body mass index; Time of MV, Time of mechanical ventilation; In-hospital max dose of metformin: In-hospital maximum dose of metformin; CLHO/HCQ: Chloroquine/hydroxychloroquine. Bold: p < 0.05

When the diabetes patients were compared by age, in Table 3 it was shown that both 29–59 and ≥ 60 groups have no significant difference in in-hospital metformin use. On the other hand, higher frequency of patients of the 29–59-group used pre-hospital metformin than patients of the ≥ 60 group (70.8 vs 56% p = 0.042). In the ≥ 60 group it was observed reduced mortality of the patients that received in-hospital metformin or pre-hospital metformin therapy than those that did not received (5.9% vs 29.2%, p = 0.001; 9.2% vs 23.5%, p = 0.035).

### CHLO/HCQ and other therapies in diabetes patients

Even though there was no significant difference in the use of vasoactive drugs, CHLO/HCQ and dexamethasone/prednisolone comparing in-hospital metformin and non-metformin users (Table 3), there was a significant difference in their outcomes. Diabetes patients that received metformin and dexamethasone/prednisolone showed reduced mortality and reduced time of insulin use than those receiving only dexamethasone/prednisolone [3.8% vs 34.6%, p = 0.004 and 3 days (1–42) vs 11 days (1–98)]. Patients that received insulin and dexamethasone/prednisolone had reduced mortality than those that only received insulin (12.1% vs 100%, p = 0.00006), however, the number of patients analyzed was low. Interestingly high mortality was observed in patients that received CHLO/HCQ than those that did not receive the therapy (32% vs 6.2%, p = 0.001). Similar result was observed for patients that received insulin therapy than those that did not receive (16.4% vs 7.1%, p = 0.047). In parallel, a higher frequency of dead patients received CHLO/HCQ, insulin or vasoactive drug compared with surviving patients (Additional file 1: Table S3).

Considering the patients that received in-hospital insulin therapy, patients from the ≥ 60-group had longer time of ICU and total time of hospitalization than those of the 29–59 group [13 days (0–94) vs 1.5 days (0–28) and 22 days (3–100) vs 9 days (4–47), p = 0.003]. Likewise, patients of the ≥ 60 group that received insulin had longer time of ICU and total hospitalization time compared to the group that did not received insulin [11.5 days (0–94) vs 2.0 days (0–37), p = 0.000]; [22 days (3–100) vs 10 days (3–70), p = 0.032, respectively) and time of mechanical ventilation [3 days (0–63) vs 0 days (0–26), p = 0.012]. Patients under insulin therapy have a more pronounced disease severity.

### Risk and protective factors for diabetes patients with COVID-19

**Fig. 1:**
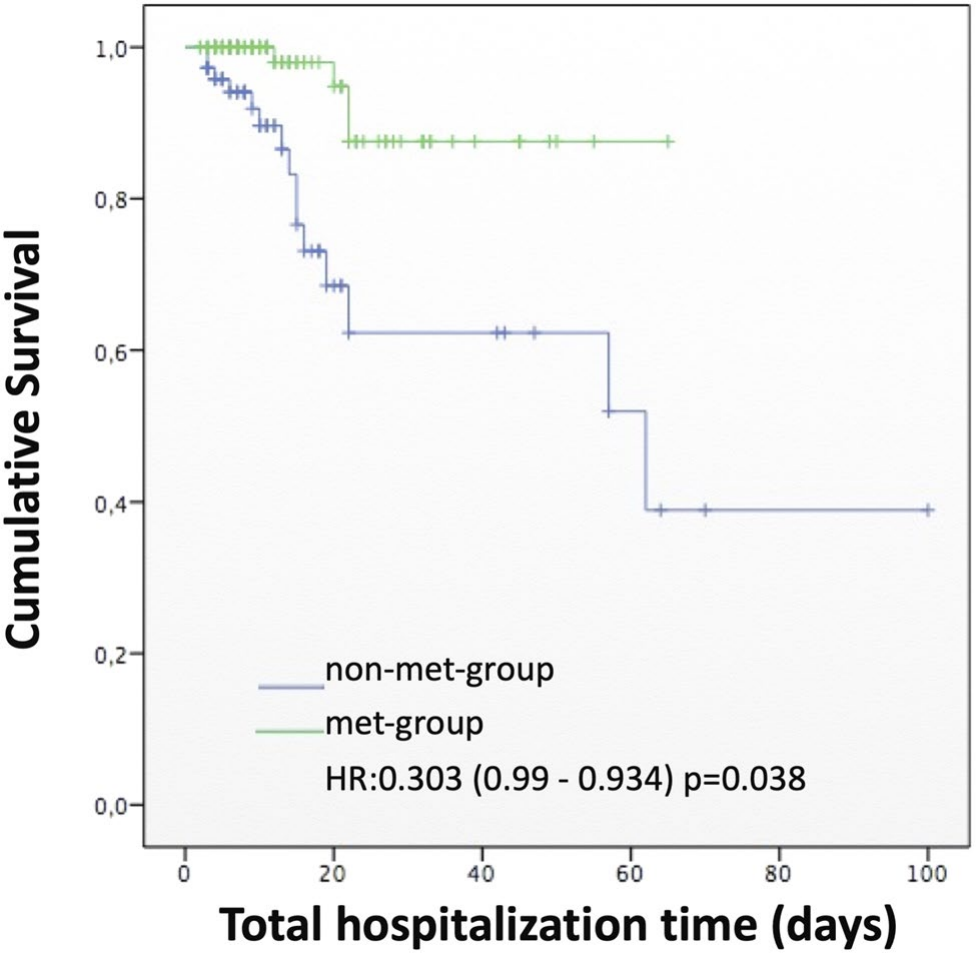
Kaplan–Meier curve for cumulative survival considering mortality during total time of hospitalization for in-hospital metformin users (met-group) and not users (non-met-group)

The univariable Cox proportional hazards regression model for in-hospital metformin therapy for diabetes patients was statistically significant [HR 0.303 (0.99–0.93), p = 0.038] and the Kaplan Meier analysis also showed that the risk of mortality was reduced in diabetes patients that received pre-hospital or in-hospital metformin (log rank p = 0.01) (Fig. 1).

Among the several risk factors for diabetes patients suffering with COVID-19, age was an important factor for severity of the disease. Univariable logistic regression showed that age was a risk factor for dying [OR 1.09 (1.045–1.14)] and orotracheal intubations [OR 1.04 (1.01–1.07)], notably higher for diabetes patients over 60 years old, with an OR of [13.04 (1.701–99.96)] for mortality and [2.65 (1.22–5.79)] for intubation. COPD/pneumonia, smoking/former smoking, neurological disease and CKD were the comorbidities associated with the risk of dying, and COPD/pneumonia, smoking/former smoking and CKD associated with intubation. Considering the in-hospital clinical characteristics, NEWS, time of stay in ICU, time of MV, need for prisma, orotracheal intubation and use of vasoactive drugs were risk factors for death. Time of stay in nursery, pre-hospital metformin therapy and in-hospital metformin therapy were good prognosis factors, reducing death risk [0.69 (0.58–0.84), 0.248 (0.089–0.685) and 0.139 (0.04–0.44)], respectively. Similar to the general group, the diabetes patients also had an increased orotracheal intubation and death risk after use of CHLO/HCQ (Additional file 1: Table S4).

All clinical characteristics, except in-hospital metformin therapy, maximum dose of in-hospital metformin, dose of pre-hospital metformin, and the use of dexamethasone/prednisolone, were risk factors for orotracheal intubation (Additional file 1: Table S4).

In the multiple linear regression including age, gender and all the comorbidities in the analysis, age was again an independent risk factor for mortality [OR 1.072 (1.003–1.15)] and for intubation [OR 1.034 (1.001–1.07)], as well as cardiopathy was a death risk factor, while CKD was an orotracheal intubation risk factor. Considering the six drugs included in this study, vasoactive drugs and CHLO/HCQ were risk factors for both outcomes (death and intubation). When considered all the in-hospital clinical characteristics, the score of clinical severities at admission (NEWS) was a risk factor for mortality, while in-hospital metformin treatment reduced the risk of mortality (Table 5).

**Table 5:** Multivariable in-hospital risk factors associated with mortality and orotracheal intubation in the diabetes patients

Characteristic	In-hospital death risk factors OR (CI 95%)	In-hospital orotracheal intubation OR (CI 95%)	Covariables included in the logistic analysis
Age Cardiopathy CDK	1.072 (1.003–1.15) 7.11 (0.16–3.98) NS	1.034 (1.001–1.068) NS 4.95–1.11–22.02)	Age and gender and all comorbidities
Vasoactive drug CLHO/HCQ	11.73 (3.07–44.87) 3.99 (1.05–15.21)	227.03 (43.19–1193-43) 31.10 (6.43–150-38)	CLHO/HCQ. In-hospital metformin therapy; Pre-hospital metformin therapy; In-hospital insulin therapy; Dexamethasone/prednisolone
NEWS In-hospital metformin therapy	1.71 (1.18–2.49) 0.034 (0.002–0.58)	NS NS	Orotracheal intubation; prona; prisma; vasoactive drug, CLHO/HCQ. NEWS, In-hospital metformin therapy; Pre-hospital metformin therapy; In-hospital insulin therapy; Dexamethasone/prednisolone

Neoplasia/immune, Neoplasia/immunosuppression; NEWS, National Early Warning Score; Prisma, prisma hemodialysis machine; Prona, prone position; CLHO/HCQ, Chloroquine/hydroxychloroquine, NS, not significant

## Discussion

In this study we attempted to contribute with information about treatment and outcome of Brazilian diabetes patients hospitalized with COVID-19, focusing on the use of metformin pre- and in-hospital, because metformin is a strong candidate for drug repurposing for treatment of COVID-19.

The results of the analysis of the complete cohort (1170 patients) clearly indicate that all the patients suffering from the examined comorbidities, that presented worse clinical features at admission or that needed more invasive therapies had also increased chance for intubation and death risk. The rate of mortality in this cohort was 4.2%, similar to the non-diabetic group (3,4%). However, in the diabetes patients it was higher (10.1%), as previously described [14]. Most deaths in both groups were among patients aged 60 years or older.

In this study age was a risk factor in univariate and multivariate analysis for mortality and orotracheal intubation in the complete cohort and in the diabetes patients. These results are in line with previous studies. Older age has been partially associated with the high frequency of multiple comorbidities [25] but it was also an independent death risk factor [26]. Age was also defined as a predictor factor for intubation [27], however in Coronado study age was not a risk factor for orotracheal intubation and/or death on day 7 [13].

As in other study [28], our data showed that diabetes patients were more likely to exhibit more comorbidities and worse clinical conditions than non-diabetes. Their intubation necessity and mortality rates were associated not only with cardiovascular and renal comorbidities but also with smoking/former smoking and neurological disease. Interestingly, in diabetes patients the CKD and the need of prisma were also risk factors for both death and intubation outcomes in univariate analysis. Moreover, CKD remains an independent intubation risk factor in multivariate analysis. In Coronado study reduced kidney function was also an independent factor of early death in diabetes patients [13], confirming the relevance of the renal function in diabetes patients with COVID-19.

In our study the use of CHLO/HCQ during hospitalization was a risk factor for intubation and death in the univariate analysis for the cohort of 1770 and for the diabetic group. In the multivariate analysis considering the in-hospital procedures and drugs, the use of CHLO/HCQ was an independent risk factor for both outcomes in the entire cohort and the diabetic group. This is a relevant information, because there is a heated debate in Brazil over chloroquine use by COVID-19 patients.

Chloroquine was proposed as an early repurposing drug against SARS-CoV-2, in vitro it has shown activity against MERS-CoV and SARS-CoV [1]. Analysis of the Cochrane Central Register of Controlled Trials (CENTRAL) indicated that use of chloroquine/hydroxychloroquine has little or no effect on the risk of death, showing increase in adverse events and having no protective effect [29]. In Brazil the use of chloroquine has been strongly recommended by the federal government, however, in this study the use of CHLO/HCQ instead of having a protective potential, was associated with a significantly increase of both the need for mechanical ventilation and death risk (Table 2). One of the concerns of chloroquine and azithromycin are their cardiotoxic potential [30]. It has been also observed that chloroquine/hydroxychloroquine increases corrected QT interval (QTc) in COVID-19 patients, however, this change was not associated with death risk [31]. In our study, even though there was no direct correlation between use of CHLO/HCQ by cardiopathic patients and increased death risk, we have observed that 50% of cardiac patients that received CHLO/HCQ died, and this rate dropped to half of it in the cardiac patients that didn’t receive the drugs during hospitalization period. Due to its pro-apoptotic activity, chloroquine has been hypothesized to select intracellular pathogens strains of SARS-CoV and HIV, that have enhanced lethality [32]. Suggesting that chloroquine could have a role in development and selection of more lethal SARS-CoV-2 strains in Brazil. Indicating that extreme caution should be taken in chloroquine or hydroxychloroquine prescription for COVID-19 patients.

It has been suggested that metformin use should be interrupted by COVID-19 patients [7], one of the main concerns was that it could cause acidosis and lactic acidosis, one report indicated that indeed patients with COVID-19 that used metformin showed an increase in acidosis and lactic acidosis (HR of 2.45 and 4.66, respectively), however, use of metformin showed no significant difference in mortality and in fact only showed significant statistical difference in reducing heart failure (HR = 0.61) and Acute Respiratory Distress Syndrome (ARDS) (HR = 0.66) [9]. So far only one preliminary report from China with a small number of subjects indicated an increase in life-threatening complications for metformin users, but with no comparison with time of hospitalization or lethality [10]. Comparison of metformin (n = 29,558) and non-metformin (10,271) users infected with SARS-CoV-2 showed that metformin is safe for COVID-19 patients [33].

Our results in this study indicated that diabetes patients that used metformin prior to admission arrived with reduced clinical severity (NEWS) and suffered less with other comorbidities. Interestingly, those patients that used metformin before hospitalization and interrupted its use had increased mortality (21.1% vs 2.1%). A study of Spanish patients with diabetes mellitus type II (DM II) and COVID-19 indicated that metformin use at home had no effect in hospital death, mechanical ventilation or time of hospitalization [24]. Data from the Health Insurance review and assessment service of South Korean from patients with COVID-19 that had DM II were divided into 3 groups (those not taking DM medication, those taking DM medications other than metformin and those taking metformin). Their conclusion was that use of insulin showed an increased hazard ratio (HR = 1.78) and that there was no correlation between clinical outcome and use of metformin. But this study only followed claims from the patients [23]. Chinese patients taking metformin prior to admission had a reduced percentage of death, however, due to lower number of patients it was not statistically significant (9.3% × 19.5%, p-value = 0.194) [14]. Other Chines study indicated that pre-metformin use associated with reduction in ICU admission in a dose-dependent way [34]. In a Belgium Centre the body mass index had no influences in outcome, while the use of metformin on admission had increased survival [15]. In French patients, the use of metformin prior to admission was lower in patients who died (OR = 0.59), while insulin was found to be associated with death of patients (OR = 1.71) [13]. Update of the Coronado study corroborates that routine metformin therapy positively associates with discharge [17]. Therefore, the data presented in this report strengthen the evidence of improved clinical features and reduced severity at admission of COVID-19 patients that use metformin before hospitalization.

Considering the use of in-hospital metformin, the results of our study showed a significantly reduction of the mortality of diabetes patients with COVID-19 (20.5% vs 3.5%). It is plausible to suppose that patients under metformin therapy were those with less severe diabetes and, thus, less at risk for severe Covid-19 infection. However, as shown in Table 3, no difference in age (the most important risk factor for death) between patients that received or not in-hospital metformin, and few clinical characteristics were more frequent in non-met-patients. Still, in patients that did not need intubation, a tendency of increased death was observed in those that did not used in-hospital metformin compared with those that received metformin. Univariate Cox proportional hazard also showed that patients that used metformin during hospitalization had reduced hazard ratio [HR 0.303 (0.99–0.93), p = 0.038]. Univariate analysis of pre- and in-hospital metformin indicated a reduced death risk [0.248 (0.089–0.685) and 0.139 (0.04–0.44)]. Multivariate analysis including all treatments revealed that use of metformin during hospitalization reduced in-hospital death [0.034 (0.002–0.58)], but not orotracheal intubation risk.

One report observed a reduction in the incidence of ARDS in Chinese patients after taking metformin during hospitalization. The 30-day mortality was 3% and 11% in the metformin and non-metformin groups, respectively. However, no difference after propensity score matching was observed (HR = 0.48) [35]. Several other different reports indicated a positive correlation with use of metformin during hospitalization and survival. A study in Italy showed that diabetes increases the risk of hospitalization and intensive care treatment. Also there was an increased risk of hospitalization in the use of insulin (OR 2.13) and a protective effect of metformin on death (OR = 0.44) [12]. Patients from Iraq that used metformin had a decreased ICU stay and time of hospitalization and decreased risk of death [20]. Russian patients that used metformin had a reduced mortality (OR = 0.26), while the ones receiving insulin had an increased mortality (OR = 2.67) [22]. Another Chinese report indicated a reduced mortality in patients that received metformin treatment (2.9% compared to 12.3% in the non-metformin group) [19]. Nursing home residents were evaluated and those that were taking metformin had a reduced hazard ratio (HR = 0.48) over the subsequent 30 days from COVID-19 diagnosis [21]. The increased death rate in patients undergoing insulin treatment may be correlated with advanced stage of diabetes [36]. Metanalysis indicated that use of metformin is associated with a reduced mortality (0.64 in a pooled adjusted model) [37, 38]. In a retrospective analysis of claims from United Health group clinical Discovery database in USA it was observed a reduction on mortality only in women that used metformin and not in men [16] and outpatient metformin therapy reduced severity of COVID-19 in adults with overweight or obesity [39]. Off-label use of metformin has been suggested for obesity treatment [40], showing also to be geroprotector and having activity against autoimmune diseases [41]. Even though metformin may have weight reduction properties, the diabetes patients with COVID-19 that were admitted in this study had similar BMI and gender distribution regardless of metformin use. BMI showed no correlation with COVID-19 severity in our cohort. Therefore, we have not observed any difference in their outcomes related to gender or BMI (data not shown) in diabetes patients under metformin therapy.

The fact that metformin has an impact in survival of COVID-19 patients, but not in orotracheal intubation indicates that metformin potential activity against COVID-19 may not be related to inhibition of viral load or pulmonary insufficiency, but instead, metformin may prevent other life-threatening conditions, like exacerbated inflammatory response. In our study, the patients that received dexamethasone/prednisolone had increased survival when this therapy was combined with metformin. Proposed mechanisms of metformin in better prognosis of COVID-19 patients are improved glucose control, reduction in body weight and insulin resistance, inhibition of viral penetration due to phosphorylation of ACE2 by AMPK, inhibition of mTor, alteration of endosomal pH, reduction of neutrophils, ROS prevention, anti-inflammatory properties, inhibition of CRAC-mediated IL6 release [42, 43]. Patients that used metformin showed reduction in pro-inflammatory markers like CRP, IL6, IL2 and TNF-α. [9]. Metformin was shown to reduce levels of IL-6 [44]. Indeed, COVID-19 patients that used metformin showed lower IL-6 levels compared to non-metformin users at admission [14]. It also may be able to promote an anti-inflammatory response through M2 polarization and decreasing IL-17 and reducing or preventing induction of cytokine storm [45]. Therefore, the use of metformin may improve clinical features of diabetes patients and reduce risk of inflammatory disease caused by COVID-19.

As a final remark is important to point some limitations of the study. It involves only one center, but it has a good test number subjects and statistical power. Still, not all drugs used by the patients pre- or in-hospital were listed, and despite our focus on metformin use one cannot exclude the influence of other treatments in the outcomes.

In conclusion, in this Brazilian single-center study, the Santa Catarina Hospital—São Paulo, diabetes was an important risk factor for COVID-19 and diabetes patients that used metformin during hospitalization showed better prognosis and reduced risk of death, metformin improves dexamethasone/prednisolone survival of COVID-19 patients. On the other hand, patients that used CHLO/HCQ have increased risk of death. This study consolidates the potential of metformin against COVID-19, but more studies as a randomized controlled study with non-diabetes patients have to be conducted to properly address this subject.

## Supplementary Information

**Additional file 1: Table S1.** Comparison of demographic and clinical characteristics of diabetes and non-diabetic patients. **Table S2.** Clinical characteristics of diabetes’ patients compared by age. **Table S3.** Clinical characteristics of diabetes patients compared between dead and non-dead patients. **Table S4.** Univariate in-hospital risk factor associated with in-hospital mortality and orotracheal intubation of diabetes patients.

## Data Availability

All data are available upon request to the authors.
