# Supplementary material for: Outcome and death risk of diabetes patients with Covid-19 receiving pre-hospital and in-hospital metformin therapies
Source: Diabetol Metab Syndr. 2021 Jul 13;13:76. doi: 10.1186/s13098-021-00695-8 (PMC8275913; doi:10.1186/s13098-021-00695-8)
Supplement: Supplementary file 1 — Additional file 1: Table S1. Comparison of demographic and clinical characteristics of diabetes and non-diabetic patients. Table S2. Clinical characteristics of diabetes’ patients compared by age. Table S3. Clinical characteristics of diabetes patients compared between dead and non-dead patients. Table S4. Univariate in-hospital risk factor associated with in-hospital mortality and orotracheal intubation of diabetes patients. [file 13098_2021_695_MOESM1_ESM.docx]

Table S1: Comparison of demographic and clinical characteristics of diabetes and non-diabetic patients

| Clinical features | Non-Diabetes  (n= 895) | Diabetes  (n= 188) | *P** | |
| --- | --- | --- | --- | --- |
| **Gender**  Man  Woman | 459 (51.3%)  436 (48.7%) | 118 (62.8%)  70 (37.2%) | | **0.005** |
| **Age, years**  Age class | 52.44 ± 15.6 | 64.59 ± 14.9 | | **0.000** |
| 29-59  ≥60 | 641 (71.6%)  254 (28.4%) | 72 (38.3%)  116 (61.7%) | | **0.000** |
| **Clinical outcome** |  |  | |  |
| Mortality  Mortality by age class  29-59  ≥60 | 30 (3.4%)  0 (0%)  30 (3.35%) | 19 (10.1%)  1 (5.5%)  18 (9.57%) | | **0.000**  0.39 |
| **Comorbidities** |  |  | |  |
| Diabetes (type I or II) | 0 (0%) | 188 (100%) | | - |
| Obesity | 87 (9.7%) | 31 (16.5%) | | **0.01** |
| Cardiopathy | 71 (7.9%) | 41 (21.8%) | | **0.000** |
| Hypertension | 241 (26.9%) | 125 (66.5%) | | **0.000** |
| COPD/pneumonia | 43 (4.8%) | 16 (8.5%) | | **0.051** |
| Smoking/former smoking | 26 (2.9%) | 13 (6.9%) | | **0.015** |
| Neurological disease | 51 (5.7%) | 13 6.0% | | 0.49 |
| CKD | 17 (1.9%) | 11 (5.9%) | | **0.005** |
| Thyroid disease | 84 (9.4%) | 24 (12.8%) | | 0.18 |
| Dyslipidemia | 100 (11.2%) | 70 (37.2%) | | **0.000** |
| Neoplasia/immuno | 44 (4.9%) | 13 (6.9%) | | 0.28 |
| Asthma | 44 (4.9%) | 3 (1.6%) | | **0.047** |
| **In-hospital clinical Characteristics** | |  | |  |
| NEWS/PEWS | 1 (0-13.2) | 2 (0-11.2) | | **0.00000** |
| Time of stay in ICU | 0 (0-107) | 3 (0.94%) | | **0.000002** |
| Time of stay in nursery | 5 (0-61) | 7 (0-58) | | **0.0001** |
| Time of hospitalization | 6 (1-112) | 10 (2-100) | | **0.000** |
| Time of MV | 0 (0-63) | 0 (0-66) | | **0.0002** |
| Orotracheal intubation | 47 (5.3%) | 45 (24.5%) | | **0.000** |
| Prisma | 13 (1.5%) | 15 (8.2%) | | **0.00001** |
| Prona | 10 (1.1%) | 7 (3.8%) | | **0.017** |
| Vasoactive drugs | 34 (3.9%) | 37 (20.1%) | | **0.000** |
| CLHO/HCQ | 53 (48.2%) | 23 (60.5%) | | 0.25 |

NS: not significant; COPD: chronic obstructive pulmonary disease; CKD: chronic kidney disease; NEWS/PEWS: National Early Warning Score / Pediatric Early Warning Score; Prisma: prisma hemodialysis machine; Prona: prone position;Neoplasia/immuno: neoplasia/immunosuppression; Time of MV: Time of mechanical ventilation; CLHO/HCQ: Chloroquine/hydroxychloroquine. Bold: p<0.05

Table S2: Clinical characteristics of diabetes’ patients compared by age.

|  | 29-59 Group  (n=72)  Number (%); | ≥60 group  (n=116)  Number (%); | *P** |
| --- | --- | --- | --- |
| **Gender**  Female  male | 25 (34.7%)  47 (65.3%) | 45 (38.8%)  71 (61.2%) | 0.57 |
| Age | - | - |  |
| Age class (years)  29-59  ≥60 | -  - | -  - |  |
| Clinical Outcome |  |  |  |
| Mortality | 1 (1.4%) | 18 (15,5%) | **0.002** |
| Mortality by gender  Female  Male |  |  |  |
| Mortality by age class  29-59  ≥60 | -  - | -  - |  |
| **Comorbidities** |  |  |  |
| Obesity | 14 (145.2%) | 17 (14.7%) | 0.39 |
| Cardiopathy | 7 (9.7%) | 34 (29.3% | **0.002** |
| Hypertension | 40 (55.6%) | 85 (73.3%) | **0.012** |
| COPD/pneumonia | 2 (2.8%) | 15 (12.9%) | **0.019** |
| Smoking/former smoking | 0 (0%) | 15 (12.9%) | **0.001** |
| Neurological disease | 1 (1.4%) | 12 (10.3%) | **0.019** |
| CKD | 2 (2.8%) | 9 (7.8%) | 0.21 |
| Thyroid | 6 (8.3%) | 18 (15.5%) | 0.18 |
| Dyslipidemia | 22 (30.5%) | 48 (41.4%) | 0.136 |
| Neoplasia/immuno | 3 (4.2%) | 9 (7.8%) | 0.38 |
| Asthma | 1 (1.4%) | 3 (2.6%) | 1 |
| BMI | 30.0 ± 5.1 | 29 ± 5.26 | 0.155 |
| **In-hospital Clinical characteristics** | |  |  |
| NEWS | 2.47 ± 2.25 | 3.49 ± 3.33 | **0.018** |
| Time of stay in ICU | 0 (0-28) | 4 (0-94) | **0.001** |
| Time of stay in nursery | 6 (0-31) | 7 (0-58) | **0.026** |
| Total hospitalization time | 7 (2-47) | 13 (3-100) | **0.000** |
| Time of MV | 0 (0-14) | 0 (0-63) | **0.001** |
| In-hospital metformin therapy | 47 (65.3%) | 68 (58.6%) | 0.36 |
| In-hospital max dose of metformin | 1253.19 ± 590.9 | 1246.32 ± 574.5 | 0.95 |
| In-hospital time of metformin therapy | 5 (1-32) | 7 (1-56) | **0.011** |
| Pre-hospital metformin therapy | 51 (70.8%) | 65 (56%) | **0.042** |
| Pre-hospital metformin daily dose | 1220.65 ± 704.6 | 1084.5 ± 547.7 | 0.28 |
| In-hospital insulin therapy | 25 (34.7%) | 36 (31.0%) | 0.63 |
| In-hospital time of insulin therapy | 5 (1-33) | 6 (1-98) | 0.13 |
| Orotracheal intubation | 10 (14.3%) | 35 (20.7%) | **0.012** |
| Prisma | 2 (2.9%) | 13 (11.4%) | **0.04** |
| Prone | 2 (2.9%) | 5 (4.4%) | 0.6 |
| Vasoactive drugs | 5 (7.1%) | 32 (28.1%) | **0.001** |
| CLHO/HCQ | 7 (9.7%) | 18 (15.8%) | 0.24 |
| Dexamethasone/prednisolone | 67 (93.1%) | 102 (87.9%) | 0.26 |

NS: not significant, Neoplasia/immune: Neoplasia/immunosuppression; BMI: body mass index; Time of MV: Time of mechanical ventilation; In-hospital max dose of metformin: In-hospital maximum dose of metformin; CLHO/HCQ: Chloroquine/hydroxychloroquine. Bold: p<0.05. Bold: p<0.05

Table S3: Clinical characteristics of diabetes patients compared between dead and non-dead patients

|  | Alive (n=169) Number (%); | Dead  (n=19) | *P** |
| --- | --- | --- | --- |
| **Gender**  Female  male | 62 (88.6 %)  107 (00.7%) | 8 42.1%  11 (57.9%) | 0.627 |
| Age | 62.97 ± 14.3 | 79.9 ± 12.5 | **0.000** |
| Age class (years)  29-59  ≥60 | 71 (42.0%)  98 (58%) | 1 (5.3%)  18 (15.5%) | **0.002** |
| Clinical Outcome |  |  |  |
| Mortality by gender  Female  Male | 62 (36.7%)  107 (63.3%) | 8 (42.1%)  11 (57.9%) | 0.643 |
| **Comorbidities** |  |  |  |
| Obesity | 27 (16.0%) | 4 (21.1%) | 0.525 |
| Cardiopathy | 29 (17.2%) | 12 (63.2%) | **0.000** |
| Hypertension | 109 (64.5%) | 16 (84.2%) | 0.123 |
| COPD/pneumonia | 11 (6.5%) | 6 (31.6%) | **0.003** |
| Smoking/former smoking | 11 (6.5%) | 4 (21.1%) | **0.05** |
| Neurological disease | 8 (4.7%) | 5 (26.3%) | **0.005** |
| CKD | 6 (3.6%) | 5 (26.3%) | **0.002** |
| Thyroid | 23 (13.6%) | 1 (5.3%) | 0.476 |
| Dyslipidemia | 63 (37.3%) | 7 (36.8%) | 0.97 |
| Neoplasia/immuno | 9 (5.3%) | 3 (15.8%) | 0.107 |
| Asthma | 3 (1.8%) | 1 (5.3%) | 0.349 |
| BMI | 29.99 ±5.08 | 28.88 ± 5.26 | 0.155 |
| **In-hospital Clinical characteristics** |  |  |  |
| NEWS | 2.62 (0-11.2) | 3.38 (0-11.1) | 0.000 |
| Time of stay in ICU | 2 (0-94) | 12 (0-58) | 0.003 |
| Time of stay in nursery | 7 (0-58) | 0 (0-18) | 0.002 |
| Total hospitalization time | 10 (2-100) | 15 (3-62) | 0.42 |
| Time of MV | 0 (0-63) | 10 (0-50) | 0.004 |
| In-hospital metformin therapy | 15 (20.5%) | 4 (3.5%) | 0.000 |
| In-hospital max dose of metformin | 1250.0±581.1 | 1225.0±585.2 | 0.93 |
| In-hospital time of metformin therapy | 8.94±9.2 | 7.0 ±10.0 | 0.68 |
| Pre-hospital metformin therapy | 110 (65.1%) | 6 (31.6%) | 0.006 |
| Pre-hospital metformin daily dose | 1144.39± 624.4 | 1380 ± 356.4 | 0.93 |
| In-hospital insulin therapy | 51 (30.2%) | 10 (52.6%) | **0.047** |
| In-hospital time of insulin therapy | 5 (1-98) | 9 (1-39) | 0.94 |
| Orotracheal intubation | 26 (17.6%) | 16 (84.2%) | **0.000** |
| Prisma | 6 (3.6% | 9 (47%) | 0.000 |
| Prone | 5 (3%) | 2 (10.5% | 1.55 |
| Vasoactive drugs | 23 (13.9%) | 14 (73.7%) | **0.000** |
| CLHO/HCQ | 17 (10.1%) | 8 (44.4%) | **0.001** |
| Dexamethasone/prednisolone | 154 (91.1%) | 15 (78.9%) | 0.107 |

NS: not significant, Neoplasia/immune: Neoplasia/immunosuppression; BMI: body mass index; Time of MV: Time of mechanical ventilation; In-hospital max dose of metformin: In-hospital maximum dose of metformin; CLHO/HCQ: Chloroquine/hydroxychloroquine. Bold: p<0.05

Table S4: Univariate in-hospital risk factor associated with in-hospital mortality and orotracheal intubation of diabetes patients

|  | Mortality Odd Ratio (CI 95%) diabetes patients | OR Orotracheal Intubation Diabetes |  |
| --- | --- | --- | --- |
| **Gender**  Female  male | 1.25 (0.48-3.29) | 0.78 (0.39-1.59) |  |
| Age | **1.09 (1.045-1.14)** | **1.04 (1.01-1.07)** |  |
| Age class (years)  29-59  ≥60 | **13.04 (1.701-99.96)** | **2.65 (1.22-5.79)** |  |
| Clinical Outcome |  |  |  |
| Mortality | - | **25.0 (6.84-91,47** |  |
| Mortality by gender  Female  Male | - | 1.55 (0.17-20.85) |  |
| Mortality by age class  29-59  ≥60 | - | 0.93 (0.03-27.9) |  |
| **Comorbidities** |  |  |  |
| Obesity | 1.40 (0.43-4.55) | 1.40 (0.59-3.34) |  |
| Cardiopathy | 8.276 (3.0-22.82) | 1.77 (0.81-3.82) |  |
| Hypertension | 2.94 (0.82-10.48) | 2.03 (0.97-4.44) |  |
| COPD/pneumonia | **6.624 (2.11-20.82)** | **3.54 (1.24-10.074)** |  |
| Smoking/former smoking | **3.83 (1.08-13.51)** | **4.76 (1.02-22.2)** |  |
| Neurological disease | **7.18 (2.07-24.93)** | 2.90(0.92-9.14) |  |
| CKD | **9.70 (2.63-35.83)** | **6.22 (1.78-22.36)** |  |
| Thyroid | 0.35 (0.045-2.77) | 0.58 (1.88-1.80) |  |
| Dyslipidemia | 0.98 (0.37-2.62) | 0.81 (0.4-1.65) |  |
| Neoplasia/immunosuppression | 3.33 (0.82-13.57) | 2.35 (0.71-7.83) |  |
| Asthma | 3.074 (0.30-31.12) | 3.18 (0.43-23.3) |  |
| BMI | 1 (0.99-1.0004) | 0.98 (0.92-1.05) |  |
| **In-hospital Clinical characteristics** |  |  |  |
| NEWS | **1.59 (1.3-1.94)** | **1.38 (1.22-1.57)** |  |
| Time of stay in ICU | **1.04 (1.00-1.07)** | **1.36 (1.23-1.49)** |  |
| Time of stay in nursery | **0.69 (0.58-0.84)** | **1.059 (1.01-1.11)** |  |
| Total hospitalization time | 1.01 (0.98-1.04) | **1.16 (1.10-1.22)** |  |
| Time of MV | **1.09 (1.03-1.15)** | - |  |
| In-hospital metformin therapy | **0.139 (0.04-0.43)** | 0.77 (0.39-1.52) |  |
| In-hospital max dose of metformin | 1.0 (0.99-1.01) | **1.1 (1.0-1.01)** |  |
| In-hospital time of metformin therapy | 0.968 (0.8-1.13) | **1.08 (1.02-1.14)** |  |
| Pre-hospital metformin therapy | **0.248 (0.09-0.68)** | 0.59 (0.29-1.16) |  |
| Pre-hospital metformin daily dose |  | 1.0 (0.99-1.01) |  |
| In-hospital insulin therapy | 2.57 (0.986-6.75) | **3.52 (1.75-7.12)** |  |
| In-hospital time of insulin therapy | 1.00 (0.962-1.041) | **1.081 (1.02-1.14)** |  |
| Orotracheal intubation | **25.01 (6.84-91.47)** | - |  |
| Prisma | **23.85 (7.08-8.03)** | **1.5 (1.22-1.84)** |  |
| Prone | 3.765 (0.678-20.91) | **1.184 (1.04-1.34)** |  |
| Vasoactive drugs | **17.287 (5.68-52.56)** | **140.12 (37-530.21)** |  |
| CLHO/HCQ | **7.11 (2.47-20.43)** | **10.23 (4.01-26.11)** |  |
| Dexamethasone/prednisolone | 0.36 (0.11-1.24) | 1.24 (0.38-3.95) |  |

Neoplasia/immune: Neoplasia/immunosuppression; COPD: chronic obstructive pulmonary disease; CKD: chronic kidney disease; NEWS/PEWS: National Early Warning Score / Pediatric Early Warning Score; Prisma: prisma hemodialysis machine; Prona: prone position; BMI: body mass index; Time of MV: Time of mechanical ventilation; In-hospital max dose of metformin: In-hospital maximum dose of metformin; CLHO/HCQ: Chloroquine/hydroxychloroquine. Bold: p<0.05
